# Exacerbation of Asthma Among Pediatric Patients Presenting to the Emergency Department

**DOI:** 10.3390/jcm14228187

**Published:** 2025-11-18

**Authors:** Karolina Pełka, Wiktoria Hanna Buzun, Jakub Dudek, Krzysztof Majcherczyk, Oliwia Klimek, Goutam Chourasia, Janusz Sokołowski, Grzegorz Gogolewski

**Affiliations:** 1 Wroclaw Medical University, 51-601 Wroclaw, Poland; karolina.pelka45@gmail.com (K.P.); goutam.chourasia@umw.edu.pl (G.C.); janusz.sokolowski@umw.edu.pl (J.S.); grzegorz.gogolewski@umw.edu.pl (G.G.); 2 Student Scientific Association of Emergency and Critical Care Medicine, Wroclaw Medical University, Borowska 213, 50-556 Wroclaw, Poland; 3 Department and Clinic of Emergency Medicine, Wroclaw Medical University, Borowska 213, 50-556 Wroclaw, Poland

**Keywords:** asthma, asthma exacerbation, asthma in paediatric patients, emergency department treatment, asthma management, GINA guidelines

## Abstract

**Background/Objectives:** Asthma exacerbations are among the most frequent causes of pediatric emergency department (ED) visits, with over 700,000 annual cases in the United States and a significant number in Europe. Children under five years of age are particularly vulnerable to hospitalization. **Methods**: As timely assessment of exacerbation severity in the ED is critical, this review synthetizes data about tools such as the Pediatric Respiratory Assessment Measure (PRAM) and the Asthma Severity Score (ASS) aid in evaluating clinical status based on respiratory rate, oxygen saturation, accessory muscle use, and response to treatment. We also analyzed the proper management following established guidelines from GINA, NAEPP and other articles. **Results**: First-line therapy includes oxygen supplementation, short-acting beta-agonists (SABAs) administered frequently during the first hour, and early systemic corticosteroids. In moderate to severe cases, ipratropium bromide is added. For refractory or life-threatening presentations, intravenous magnesium sulfate, epinephrine, or ventilatory support may be required. Discharge is appropriate when symptoms resolve, oxygen saturation remains >94% on room air, and the child demonstrates adequate inhaler use. Hospitalization is indicated in cases of persistent hypoxemia, poor response, feeding difficulties, or social concerns. Post-discharge care includes thorough caregiver education, medication access, and a personalized asthma action plan to reduce recurrence risk. **Conclusions**: The effective diagnosis, appropriate exacerbation treatment, monitoring of patients in the post-attack period, as well as successful preventive medication play a key role in the management of pediatric patients with asthma.

## 1. Introduction

Bronchial asthma is the most common chronic respiratory disease in children, affecting over six million pediatric patients in the United States and a significant number in Europe [1]. According to the Global Initiative for Asthma (GINA), asthma is defined as a chronic inflammatory disease of the airways characterized by variable respiratory symptoms and reversible airflow limitation [2,3]. Airway inflammation involves activation of immune and structural cells leading to mucosal edema, mucus hypersecretion and bronchial hyperresponsiveness [4].

**Figure 1:**
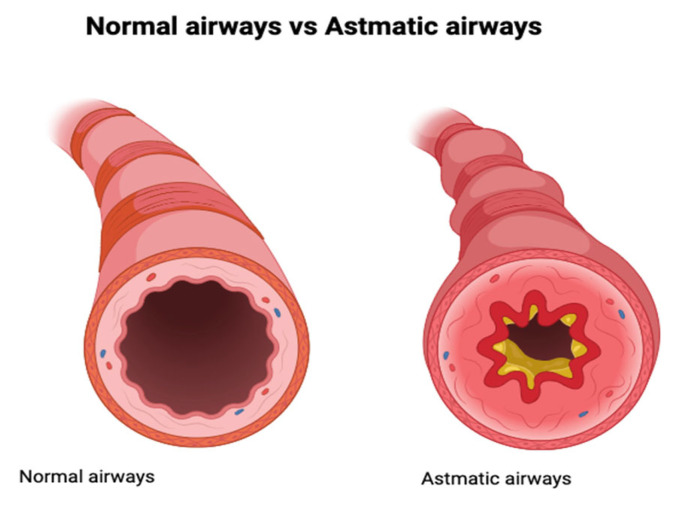
Comparison of normal airways to airways during asthma exacerbation [6].

A major structural consequence of ongoing airway inflammation is airway remodeling, which includes subepithelial fibrosis, increased smooth muscle mass, goblet cell hyperplasia and thickening of the basement membrane [5]. These changes may develop early in childhood, even in the absence of clinically apparent inflammation, suggesting the involvement of epithelial–mesenchymal interactions during disease development [4,5,6]. The structural differences between normal airways and those during an asthma exacerbation are illustrated in Figure 1 [7].

The concept of asthma control refers to minimizing symptoms, functional limitations and the risk of future exacerbations through appropriate therapy [2]. In contrast, an asthma exacerbation is defined as an acute or subacute worsening of symptoms such as dyspnea, wheezing, cough and chest tightness requiring a change in treatment [2,8].

Emerging evidence highlights the key role of the small airways (airways <2 mm in diameter) in pediatric asthma. Dysfunction in the small airways contributes to poor symptom control, increased disease severity and a higher risk of exacerbations [6]. Their involvement has therefore become clinically relevant when assessing disease severity and treatment response.

Asthma is one of the most common chronic diseases in children worldwide. The prevalence of childhood asthma is influenced by genetic susceptibility, environmental exposures and socioeconomic factors [1,9]. Comorbidities such as allergic rhinitis, atopic dermatitis, food allergies and obesity are frequently present and may negatively affect asthma control and outcomes [4]. Asthma exacerbations occur most frequently in preschool-aged children, particularly in association with viral respiratory infections, and remain one of the leading causes of emergency department (ED) visits and hospital admissions in this age group [8].

In the ED, rapid severity assessment and prompt initiation of treatment based on current recommendations, including those from GINA and the National Asthma Education and Prevention Program (NAEPP), are essential to prevent clinical deterioration and improve outcomes [2,10].

## 2. Asthma in Children—Epidemiology and Risk Factors

### 2.1. Epidemiology of Asthma—Incidence, Prevalence and Comorbidities

Genetic and environmental factors, as well as maturation of the immune system in the early period of life, may contribute to the occurrence of the disease, with large discrepancies across time and geographic regions [10]. In 2021, the global prevalence of childhood asthma reached 95.7 million cases, with the Low SDI region recording 25.4 million cases. In the case of asthma and atopic dermatitis (AD), the global prevalence amounted to 72.4 million cases, mainly in the Middle SDI regions (19.7 million cases). Between 1990 and 2021, age-standardized incidence rates declined for both conditions. Geographic differences were noticeable: high-income North America demonstrated the highest prevalence of asthma, whereas Western Europe was the leader in terms of AD prevalence. The global DALY index associated with asthma decreased from 6.9 million in 1990 to 4.6 million in 2021, with significant regional differences [11]. In the meta-analysis by Zhou et al., 164 studies including 1,547,404 children were analyzed. The overall prevalence of childhood asthma was 10.2%. The highest incidence rate was in Oceania (23%), and the lowest in Eurasia (8%) [12]. It is worth noting the issue of the frequency of asthma exacerbations in children. In the study by Hurst et al., a total of 5045 asthma exacerbations were recorded, corresponding to 0.27 exacerbations per patient per year (27.56 per 100 person-years). The significance of asthma exacerbations in the pediatric population is underscored by the fact that as many as 37% of children experienced at least one exacerbation during the study period [13].

Mendy et al., after analyzing data from 27,437 participants, found that the age at which asthma develops appears to be a key factor determining the phenotype of the disease. It was shown that adult-onset asthma is associated with a higher likelihood of coexisting obesity, hypercholesterolemia, hypertriglyceridemia, osteoarthritis, diabetes mellitus, and hypertension. In contrast, an earlier age at asthma diagnosis is associated with an increased probability of developing chronic obstructive pulmonary disease (COPD) [14].

### 2.2. Risk Factors for Asthma

In the meta-analysis by Zhou et al., risk factors for childhood asthma were also comprehensively identified, which included older age, male sex, obesity, parental smoking, high maternal education, preterm birth, cesarean section, lack of breastfeeding, family history of asthma, rhinitis, eczema, pets, high traffic density, meat, margarine, fast foods, use of paracetamol, and use of antibiotics [12].

In the subsequent meta-analysis, Sio Y. et al. focused on risk factors for asthma in the Asian population. A total of 289 scientific articles were analyzed. Among the most frequently reported risk factors were: family history related to allergy (family history of asthma, family atopic history, family history of any allergic diseases, family history of allergic rhinitis, family history of atopic dermatitis), factors related to housing conditions (presence of mold, cockroaches, water damage, and incense burning), exposure to tobacco smoke, active cigarette smoking, parameters related to body mass index (BMI), various types of air pollution, and pre- and perinatal factors (low birth weight, preterm delivery, cesarean section) [15].

In the population-based study, Osvald et al. demonstrated that asthma in children and young adults is associated with higher mortality, regardless of the presence of life-limiting disease or the socioeconomic status of the parents [16].

In the study by Frey et al., it is also emphasized that the prevalence of childhood asthma in the United States, measured in the National Health Interview Survey (NHIS), decreased by 30% since 2017. After the onset of the pandemic, a noticeable lack of increase in the number of visits to the pediatric emergency department due to asthma and only a limited increase in the number of hospitalizations were also recorded. The authors, however, emphasize that this apparent decline may have negative long-term consequences, since the change in asthma risk factors during the pandemic (exposure to rhinovirus infections and allergic sensitizations) may adversely affect the prevalence of the disease in the future [17].

## 3. Assessment of the Severity of Asthma Exacerbations in Children in the Emergency Department

The diagnosis of asthma exacerbation in children is primarily based on clinical assessment, including evaluation of body position, speech, level of consciousness, respiratory rate, use of accessory muscles, heart rate, oxygen saturation, and peak expiratory flow (PEF). This allows for the identification of three severity levels of asthma exacerbation in children: mild/moderate, severe, and life-threatening. The assessment criteria are presented in Table 1 and Table 2 [3].

### 3.1. The Pediatric Respiratory Assessment Measure (PRAM)

For the assessment of the severity of asthma exacerbations in children, many useful tools are applied. The most important of them is The Pediatric Respiratory Assessment Measure (PRAM) (Table 3). PRAM is a tool utilizing 5 assessed parameters such as: presence of intercostal retractions, presence of suprasternal retractions, presence of wheezing, presence of vesicular breath sounds over the lung fields, and assessment of SpO_2_ saturation [18,19,20]. This scale was described and validated already in 2008 by Ducharme et al., who reported the validity, responsiveness, and reliability of PRAM as an extremely effective tool in assessing the severity of asthma exacerbation in children aged 2 to 17 years with acute asthma [18].

In the study by Douros et al., children aged 3–16 years with mild to moderate asthma exacerbations participated. All children received 1 mg/kg body weight of oral prednisolone and 4–6 puffs of salbutamol via MDI/S inhaler. The aim of the study was to estimate the risk of hospitalization in children, assessed using the PRAM score. Hospital admission was positively associated with the initial PRAM score (OR: 18.91, CI: 2.42–123.12, *p* = 0.005) and negatively with improvement in the PRAM score (OR: 0.52, CI: 0.01–0.78, *p* = 0.032). This confirms that the PRAM tool is reliable and can be effectively used to estimate the severity of asthma exacerbation [21]. When discussing PRAM, it is worth noting the AAIRS scale, a 17-point (0–16) derivative of PRAM, developed to enable better differentiation for research purposes [22].

### 3.2. The Pediatric Asthma Severity Score (PASS) and the Modified Pulmonary Index Score (MPIS)

When reviewing the literature on other tools for assessing asthma exacerbation in children, it is worth focusing on the Pediatric Asthma Severity Score (PASS) (Table 4) and the Modified Pulmonary Index Score (MPIS) (Table 5) [23,24]. In the study by Ryan et al., correlations were described between the PASS in children prior to admission and the risk of subsequent deterioration and the need for later hospitalization. The study indicates that a PASS value ≥ 9 identifies children at increased risk of subsequent emergency admissions and readmissions to the pediatric intensive care unit. PASS is a tool that can assist in the qualification of asthma exacerbation in children and in making decisions regarding hospitalization [23].

In the study by Miller et al., the association between the Modified Pulmonary Index Score (MPIS) and the predicted length of stay in the pediatric intensive care unit was described. After accounting for demographic data and medical history, the length of stay in the pediatric intensive care unit was 2.09 times longer (MPIS 6–9) and 2.68 times longer (MPIS ≥ 10) compared to children with an MPIS < 6. In another study, Okada et al. also demonstrated that a therapeutic strategy based on MPIS, by enabling caregivers to more accurately assess asthma exacerbation, may shorten hospital stay. In summary, higher MPIS was associated with longer hospital stay and longer duration of continuous albuterol administration [24,25,26,27].

**Table 5:** Modified Pulmonary Index Score (MPIS) [27].

Score	SpO_2_ Saturation Index	Accessory Muscle Use	I:E Ratio	Wheezing	Heart Rate	Respiratory Rate
0	≥95%	none	2:1	none	<3 years: <120 ≥3 years: <100	<6 years: ≤30 ≥6 years: ≤20
1	93–95%	mild	1:1	end-expiratory wheezes	<3 years: 120–140 ≥3 years: 100–120	<6 years: 31–45 ≥6 years: 21–45
2	90–92%	moderate	1:2	inspiratory and expiratory wheezes, with good air entry	<3 years: 141–160 ≥3 years: 121–140	<6 years: 46–60 ≥6 years: 36–50
3	<90%	severe	1:3	inspiratory and expiratory wheezes, with reduced air entry	<3 years: >160 ≥3 years: >140	<6 years: >60 ≥6 years: >50

### 3.3. The Asthma Clinical Score (ACS)

Another noteworthy scale is the Asthma Clinical Score (ACS) (Table 6). In the study by Andoh et al., the usefulness of assessing asthma exacerbation using the ACS was described. It demonstrated a better ability to detect response than PRAM (31% vs. 15%, respectively). Pre-treatment ACS showed predictive validity comparable to PRAM. This study confirmed that ACS is a useful tool for assessing the severity of asthma exacerbations in children in the hospital emergency department (ED) [28].

In summary, all of the above-mentioned scales have been investigated for their usefulness in clinical practice and are successfully used in emergency department settings (ED) [20].

## 4. Treatment of Asthma Exacerbation in Children in the Emergency Department

Severe asthma exacerbation is considered a life-threatening condition, therefore patients presenting with acute asthma should be treated in a hospital setting, such as a hospital emergency department (ED). Furthermore, in the most severe cases, this condition can lead to respiratory failure requiring ventilation support as well as treatment in the intensive care unit (ICU) [29]. Hospitalization of children with asthma exacerbation is mainly associated with adverse clinical indicators in the emergency department and the need for intubation due to a history of asthma [30]. The basis for effective treatment is a rapid and accurate assessment of the severity of the exacerbation [31]. According to the 2025 GINA guidelines, treatment methods for pediatric patients have been divided according to age- “children under 5 years of age” and “children aged 6–11 years, adolescents and adults” [3].

### 4.1. Primary Medications Used in the Treatment of Asthma Exacerbations in the ED

For children with asthma exacerbation in the emergency department, treatment include: oxygen therapy, inhaled short-acting beta2-mimetics, a combination of inhaled corticosteroids (ICS) and formoterol, epinephrine, systemic corticosteroids, and inhaled corticosteroids. In order to achieve rapid improvement in the patient’s condition, these methods are usually used simultaneously [3]. The particular doses of the medication listed above are presented in Table 7. The classification of drug groups used in asthma exacerbations and chronic treatment is presented in Table 8.

#### 4.1.1. Oxygen Therapy

The correct arterial blood oxygen saturation in children is ≥94%. Hypoxemia should be treated immediately by administering oxygen through a cannula or oxygen mask. Studies have shown that administering high concentrations of oxygen may adversely affect carbon dioxide elimination [32]. This condition increases the risk of developing respiratory failure, which is considered a life-threatening symptom of asthma. For this reason, it is recommended to use a titrated oxygen regime in patients with acute asthma exacerbation, optimized to the patient’s clinical condition [33]. To avoid deterioration of blood oxygenation in children who experience particular discomfort when changing medications, oxygen therapy should be administered immediately in combination with SABA (2.5 mg or dissolved in 0.9% NaCl) administered via an oxygen nebulizer [34].

#### 4.1.2. Short-Acting Beta2-Mimetics (SABA)

Currently, salbutamol is the primary pharmaceutical (alternatively albuterol), in this group of drugs, used to treat acute asthma. The most effective way to administer the drug is to use a pressurized Metered Dose Inhaler (pMDI) with a spacer [35,36,37]. This reduces the risk of complications from the drug settling in the mouth (e.g., thrush) and increases the amount of drug reaching the lungs [38,39]. The alternative way to administer medication is an air-driven nebulizer. However, nebulizers are reported to be associated with a risk of transmitting infectious particles.

Routine use of intravenous beta-agonists in most patients with severe asthma exacerbation is not recommended due to a lack of evidence of their effectiveness. For example, in a study by Travers A.A. et al. (2001), among 584 patients, no subgroups were identified in which intravenous administration of beta-2 agonists resulted in clinical improvement [40].

It has been proven that its introduction into therapy is associated with a 27% decrease in hospitalizations compared to the use of SABA alone in children with exacerbation in the ED. In addition, chronic nebulization, continuous administration of magnesium sulfate, and the use of levosalbutamol are not recommended [3,41].

#### 4.1.3. Combination ICS/Formoterol in Children Aged 6–11 Years Old, Adolescents and Adults

Low doses of ICS/formoterol have been shown to have a similar efficacy and safety profile to high doses of SABA [42]. A randomized study by Jonkers R.E. (2006) found single doses of budesonide/formoterol to be effective in patients with experimentally induced bronchial obstruction [43]. The administration of ICS and long-acting beta2-mimetics (LABA) using a single inhaler results in their synergistic action [44]. Improvement in the patient’s clinical condition is observed as early as 1 min after inhalation of the budesonide/formoterol combination [45,46].

The efficacy of salbutamol and formoterol administered together with ICS was also compared in children aged 5 to 15 years, where it was shown that both drugs have a similar beneficial bronchodilator effect and that budesonide/formoterol alone can be used in children with mild acute asthma exacerbation [47]. In addition, it has been proven that the use of budesonide/formoterol in patients undergoing maintenance treatment is an effective method of protection against severe asthma exacerbations [48].

#### 4.1.4. Epinephrine in Children Aged 6–11 Years Old, Adolescents and Adults

Epinephrine is not recommended for the treatment of asthma exacerbations [49]. It is acceptable to use it as an adjunct to basic treatment in patients with asthma exacerbations associated with anaphylaxis or vasovagal shock [50].

#### 4.1.5. Systemic Corticosteroids (SCS)

Systemic corticosteroids should be used routinely in acute medical care settings for patients with any exacerbation of asthma, except for the mildest [51]. Specific indications include patients whose condition has not improved after treatment with SABA, patients whose exacerbation developed while taking oral corticosteroids (OCS), and those who have required OCS in the past.

The most effective treatment is to administer the drug within one hour of the onset of exacerbation symptoms. Both oral and intravenous administration have similar efficacy, but liquid preparations are preferred over tablets in pediatric patients [52].

Furthermore, a randomized study by Cronin J.J. et al. (2015) demonstrated that a single dose of oral dexamethasone (0.3 mg/kg) is as effective as a 3-day course of oral prednisolone (1 mg/kg daily) [53]. No differences in the effects of these preparations were also found among emergency department patients in a study by Dahan E. et al. (2022) [54]. A one- or two-day course of dexamethasone should therefore be considered as an alternative to a 3–5-day course of prednisolone [55,56]. Oral dexamethasone should not be used for more than 2 days due to metabolic side effects. If there is no improvement, modification of treatment and administration of prednisolone should be considered [57].

SCS have been proven to reduce the risk of hospitalization in patients admitted to the ED with asthma exacerbation. The benefits appear to be greatest in patients with more severe asthma and in those who are not currently receiving steroids [58]. Despite the lack of clear data indicating the superiority of intramuscular glucocorticosteroids over oral administration, children discharged home from the ED receive an intramuscular dose of CS as a preventive measure.

#### 4.1.6. Inhaled Corticosteroids (ICS)

The use of systemic corticosteroids is crucial in the treatment of asthma exacerbations. However, studies indicate that the use of inhaled corticosteroids may also be beneficial, as they reduce the risk of SCS side effects [59]. The use of ICS in pediatric patients within the first hour of admission to the emergency department reduces the chance of hospitalization among patients who have not been treated with oral or intravenous corticosteroids [60]. Similar results were obtained for patients who received high doses of ICS in combination therapy with SCS [61].

For children under 5 years of age the use of inhaled corticosteroids is only indicated to shorten the hospital stay and improve the clinical condition of patients with severe asthma attacks. Budesonide is usually used as an inhaled CS. This treatment should be reserved for individual cases [62,63]. A study conducted by B.H. Rowe et al. also found that patients discharged from the ED after treatment for acute asthma exacerbation benefit from additional treatment with high doses of inhaled budesonide for 21 days compared to oral corticosteroids alone [64].

### 4.2. Other Therapeutic Options in Children Aged 6–11 Years Old, Adolescents and Adults

#### 4.2.1. Ipratropium Bromide

The addition of ipratropium bromide to beta2-mimetics slightly improves the clinical condition of patients, especially among children with severe asthma exacerbation [65,66]. In addition, a study by Craig S.S et al. (2020) showed that the combination of inhaled anticholinergic drugs and inhaled beta2-agonists reduced the risk of hospitalization in these patients [67].

#### 4.2.2. Magnesium Sulfate

It is not recommended for routine use in patients with exacerbated asthma. It may be beneficial in patients with severe exacerbations, as an adjunct to inhaled beta2-mimetics, causing a slight improvement in lung function [68,69,70,71].

For children under 5 years of age a single intravenous dose of magnesium sulfate is recommended as an adjunct to treatment after the first hour of standard therapy in children ≥2 years of age with severe asthma attacks. Its effect on improving lung function (PEFR) has been proven only when administered intravenously, not when nebulized [68,72,73].

#### 4.2.3. Helium Oxygen Therapy

There is evidence in favor of heliox (helium oxygen therapy). Studies confirm the positive effect of adding heliox to nebulized beta2-agonists in patients with acute asthma [74,75]. In a prospective randomized study by Rose J.S. et al. (2002), no improvement in FEV1 was demonstrated 2 h after the use of heliox-powered nebulizers, but patients reported an improvement in perceived dyspnea [76].

#### 4.2.4. Leukotriene Receptor Antagonists (LTRAs)

Anti-leukotriene drugs are used as adjunctive therapy in chronic asthma. Their effectiveness in the treatment of acute asthma has not been fully confirmed [77,78]. A 2008 study conducted by Nelson K.A. et al. indicates no improvement in FEV1 within 3 h of oral montelukast administration in children aged 6–14 years with exacerbated asthma [79]. However, in 2012, Hermanci K. et al. demonstrated the beneficial effect of oral montelukast (4 mg) when administered in combination with SABA [80].

#### 4.2.5. Non-Invasive Ventilation (NIV)

The use of NIV in patients with severe asthma can accelerate the improvement of lung function and shorten the period of hospitalization [81]. Although this method is routinely used in clinical practice, there is insufficient evidence to recommend it [82,83].

### 4.3. Not Recommended Medications in Children Aged 6–11 Years Old, Adolescents and Adults

In the event of asthma exacerbation, theophylline, antibiotics, and sedatives are not recommended.

Theophylline is not recommended due to its poor therapeutic benefits and high risk of adverse effects, such as gastrointestinal disorders, serious heart rhythm disorders, and seizures [84]. Therefore, theophylline therapy should be avoided when other, more effective and safer treatments are available [85].

Antibiotics should only be used when there is strong evidence of respiratory tract infection. There is limited evidence that antibiotics have a beneficial effect in the treatment of asthma [86,87].

Sedatives can cause respiratory depression, which can lead to death in patients with severe asthma exacerbation [88,89]. In addition, there is evidence that patients using sedatives are more prone to serious complications of asthma [90].

**Table 7:** Doses of medication used in ED asthma management [3].

	Under 5 Years of Age	Children Aged 6–11 Years
Oxygen	Maintain ≥ 94% To avoid deterioration of blood oxygenation it could be combined with 2.5 mg SABA (or dissolved in 0.9% NaCl).	Maintain ≥ 94%
SABA (salbutamol)	Via pMDI 4 inhalations (100 µg per puff; in severe asthma 6 puffs); Via nebulizer the dose should be 2.5 mg; Further dosing is decided based on the patient’s clinical condition during observation [91].	Via pMDI 4 to 10 inhalations (100 µg per puff) every 20 min for 1 h; After first hour it can be repeated every 3–4 h or 6–10 inhalations every 1–2 h; Via nebulizer the dose should be 2.5–5 mg for 30 min. It can be repeated up to (max) 4 doses per day [92].
SCS	For methylprednisolone it is 1–2 mg/kg/day (max 20 mg/day for children < 2 years and 30 mg/day for children 2–5 years) for 3–5 days [52,93]; An alternative to methylprednisolone is dexamethasone in a single dose of 0.3–0.6 mg/kg (max. 12 mg) [54].	For prednisolone it is 1–2 mg/kg up to a maximum of 40 mg/day for 3–5 days [94].

**Table 8:** Asthma medication classification: drugs used in asthma exacerbation and those used in chronic treatment [3].

Reliever Medication	Maintenance Treatment
**Anti-inflammatory Reliever Medication**	Inhaled corticosteroids (ICS) ICS in combination with SABA Leukotriene receptors agonists (LTRA) and leukotriene modifiers
Low dose combination ICS-formoterol Low dose combination ICS-SABA	
**Short-acting Bronchodilator Reliever Medication**	**Add-on maintenance medications**
Short-acting inhaled beta2-agonist bronchodilator (SABA) Short-acting antimuscarinics (anticholinergics)	Long-acting muscarinic agonists (LAMA) Anti-IgE Anti-IL5 and Anti-IL5Ralfa Anti-IL4Ralfa Anti-TSLP Systemic corticosteroids

## 5. Treatment Algorithm for Asthma Exacerbations According to Severity

### 5.1. Management in Children Aged 6–11 Years, Adolescents and Adults

#### 5.1.1. Mild/Moderate Exacerbation

In the case of mild/moderate asthma exacerbation, a patient presenting to the emergency department should remain there for at least one hour after the start of treatment. Treatment can be administered in the ED and consists of maintaining oxygen saturation at ≥94% and administering salbutamol (4–10 puffs by pMDI with spacer every 20 min). Depending on the patient’s clinical condition, especially if the exacerbation is classified as moderate, the addition of ipratropium bromide or high doses of ISC in patients who are not receiving SCS, should be considered [95].

#### 5.1.2. Severe Exacerbation

For patients with severe exacerbation, treatment includes oxygen and SABA, as well as ipratropium bromide and oral (prednisolone) or intravenous corticosteroids. In addition, intravenous magnesium should be considered in some patients.

In both cases the patient’s condition should be monitored in order to quickly recognize any deterioration. Treatment should be started within the first hour after admission to ED. After one hour, lung function should be measured and, depending on the result, the patient should be discharged home with recommendations or continue treatment in the ED [31].

#### 5.1.3. Life-Threatening Exacerbation

Life-threatening asthma exacerbation often requires treatment in an ICU, so the patient’s condition and the need for transfer to the ICU must be assessed quickly. In the emergency department, treatment includes oxygen therapy, SABA, securing the airways (a ventilator may be necessary), and arterial blood sampling for blood gas analysis. If a patient requires transfer to the ICU, they should be stabilized and monitored during the transfer [96].

**Figure 2:**
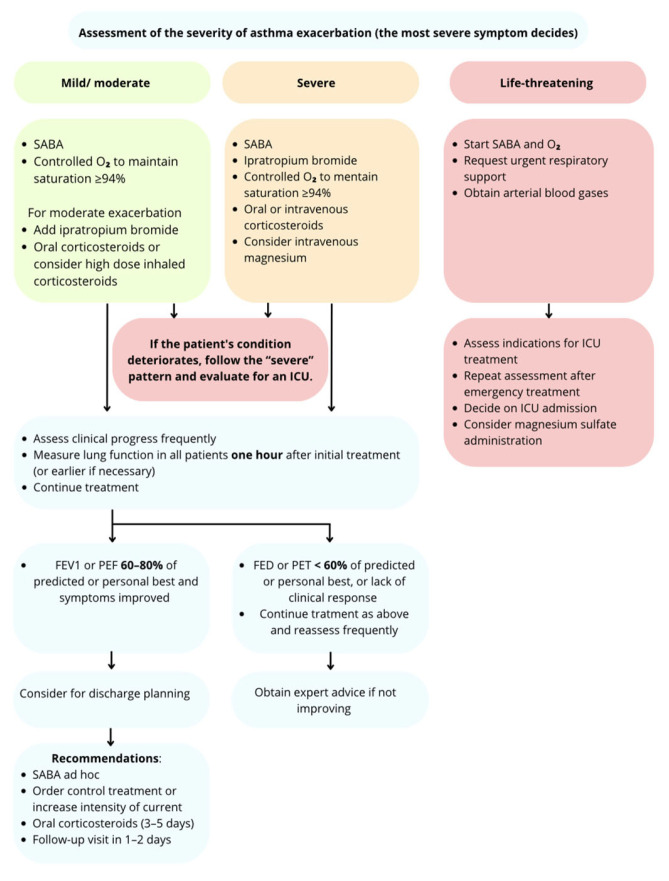
Management depending on the severity of asthma exacerbation in children ≥6 years of age and adolescents [3,92].

The management depending on the severity of exacerbation in children aged 6–11 years and adolescents is presented in Figure 2.

### 5.2. Management Depending on the Severity of Exacerbation of Asthma in Children Under 5 Years of Age

#### 5.2.1. Mild/Moderate Exacerbation

In patients with exacerbations classified as mild or moderate, treatment in the ER begins with the administration of salbutamol (or albuterol) at a dose of 4 × 100 qg administered using a pressurized metered-dose inhaler (pMDI) with an inhalation chamber or a first dose of 2.5 mg in nebulization [97]. It is not recommended to use subsequent doses of SABA if a good and long-lasting response is observed (PEF > 60–80%; 3–4 h). In oxygen therapy, we aim to achieve saturation ≥ 94% [98]. Additionally, in moderate exacerbations, consider continuing salbutamol at the above doses, twice every 20 min if needed, and adding ipratropium bromide and oral glucocorticosteroids to the treatment. In children with an insufficient response to SABA or moderate to severe exacerbation, it is recommended to add ipratropium bromide to the therapy at a dose of 4 puffs of 20 mcg per puff or 250 mcg by nebulizer every 20 min in 3 doses. Next, intensive monitoring of the patient in the ICU for 1–2 h is recommended, and in the absence of response to salbutamol or continued symptoms of exacerbation, another 4 doses of salbutamol should be administered or ipratropium bromide or OCS should be added if not already given. In case of deterioration or lack of improvement, proceed as in severe exacerbation [3,99,100].

#### 5.2.2. Severe Exacerbation

In patients whose condition is classified as severe asthma exacerbation, administer 100 µg of salbutamol in 6 puffs using an oxygen-powered nebulizer every 20 min. Additionally, use ipratropium bromide in nebulization and OCS [92]. Oxygen therapy to maintain saturation ≥94% [101]. Intravenous magnesium sulfate should be considered [97,102].

**Figure 3:**
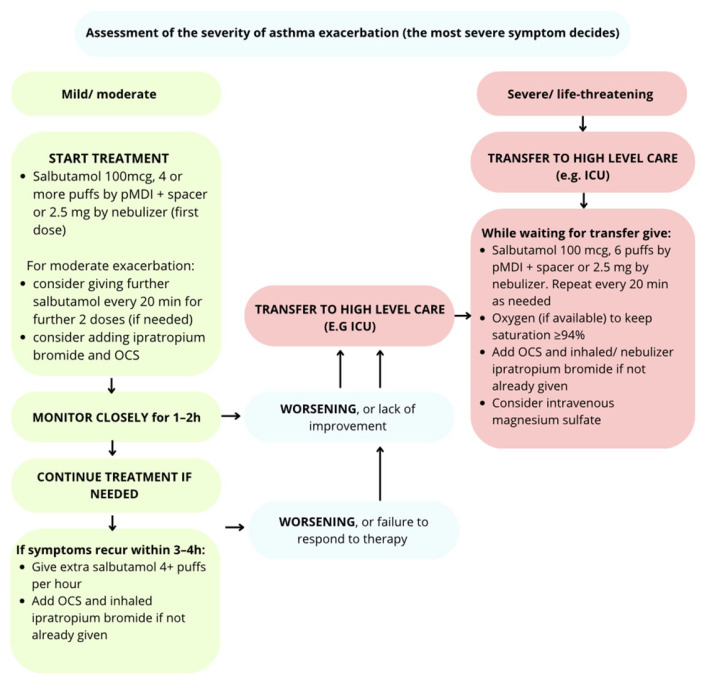
Management depending on the severity of asthma exacerbation in children <5 years of age [3,92].

The management depending on the severity of exacerbation in children under 5 years of age is presented in Figure 3.

## 6. Criteria for Hospitalization and Discharge from the Emergency Department

Hospital admission for pediatric patients experiencing asthma exacerbation and presenting to the Emergency Department (ED) follows well-defined standards and treatment algorithms. Although specific protocols may vary slightly, several key diagnostic criteria are widely applied to guide both admission and subsequent management [103]. Central considerations include assessment of the patient’s clinical severity, therapeutic response, and risk factors precipitating asthma attacks. Prompt diagnosis and initiation of therapy within the first “golden hour” are crucial for optimal prognosis [104]. In order to admit a child with worsening asthma to the emergency department, they must first meet criteria such as: CRS ≥ 3 despite adequate therapy (Clinical Respiratory Score based on assessment of respiratory rate, auscultation, use of accessory breathing muscles, mental status, Room Air SpO_2_ and color of the patient) and O_2_ requirement to keep SpO_2_ > 90% [103,105].

Hospitalization rates rise significantly in children with oxygen saturation below 94%, respiratory rates exceeding 31 breaths per minute, a history of pneumonia, atopic dermatitis, poorly controlled asthma requiring more than six SABA (short-acting beta-agonist) inhalers over 12 months, recent ED visits or hospitalization, and a family history of atopic disease (asthma, allergic rhinitis, or atopic dermatitis) [106].

On presentation, the primary evaluation focuses on asthma severity. Key parameters include respiratory effort, oxygen saturation, respiratory rate, and heart rate. Observation of the child’s posture is important—whether the patient can lie flat or prefers sitting upright, presence of diaphoresis, use of accessory respiratory muscles, wheezing, or a “silent chest” [107]. Guidelines developed by the Global Initiative for Asthma (GINA) help classify severity and urgency, shaping subsequent ED management and pacing [30]. In practice, the *Preschool Respiratory Assessment Measure* (PRAM) is also employed to assess bronchial asthma exacerbations in children aged 3–6 years. PRAM evaluates five clinical parameters—use of accessory muscles, suprasternal retractions, prolonged expiration, wheezing, and oxygen saturation—scored from 0 to 2 or 0 to 3 points, with a maximum of 12 [108]. Higher scores indicate greater airway obstruction and more severe exacerbation, enabling standardized, accurate assessment and expedited decision-making consistent with GINA guidelines [109].

Initial management of asthma patients aims to rapidly reverse airway obstruction through timely medical interventions and correction of associated disturbances such as hypoxemia, hypercapnia, or electrolyte imbalance. Clinicians must remain vigilant for potential complications, including hypotension, hyperventilation, or pneumothorax, and continuously monitor the patient’s evolving status [29]. Non-invasive ventilation (NIV) is increasingly favored due to lower costs and shorter hospital stays compared with invasive mechanical ventilation (IMV). Conventional endotracheal intubation is now largely reserved for critically ill patients with absolute indications but it is still quite popular in many facilities. However, reducing the need for its use shows a number of clinical benefits and safety advantages, as well as growing acceptance of NIV [110,111].

During hospitalization, first-line therapy consists of an inhaled short-acting beta-agonist (SABA), such as salbutamol, appropriate even for children over two years old. In moderate to severe exacerbations, systemic corticosteroids and ipratropium bromide—often administered in the first hours in combination with SABA—should be considered to reduce hospitalization rates and restore pulmonary function. Continuous monitoring is essential to evaluate therapeutic effectiveness and detect complications [112].

In severe cases unresponsive to ED management, patients are transferred to other hospital wards, in accordance with the hospital’s administrative procedures, for example, to the higher-level inpatient care like a Pediatric Intensive Care Unit (PICU). Admission criteria there include: SABA administration beyond four hours with persistent symptoms, inadequate ventilation with hypercapnia (capillary blood PCO_2_ > 45 mmHg), need for high-flow nasal cannula or noninvasive ventilation, persistent hypoxemia (SpO_2_ < 90%) despite supplemental oxygen (>2 L/min or >50% FiO_2_ via non-rebreather mask), or altered mental status (somnolence) [113]. Transfer is also appropriate when no clinical improvement or worsening respiratory failure occurs despite maximal possible therapy and the use of all available measures in the emergency department.

Discharge is appropriate when core criteria are met: SABA needed at intervals longer than four hours after 4–8 h of treatment, adequate oxygen saturation without ventilatory support, minimal respiratory distress, improved pulmonary function tests (PFTs), ability to eat and ambulate normally, and a safe plan for home monitoring [114]. In order for a child with worsening asthma to be discharged from the emergency department, the course of their illness must be normalized. Asthma must return to remission and control, without causing any problems in daily functioning. Discharge can be carried out by paying attention to basic criteria, such as: CRS 0–2, easy work of breathing with good air exchange, SpO_2_ > 90% on room air consistently, able to maintain O_2_ sats, RR, WOB (Work of Breathing) during feeding/activity and family verbalizes ability to manage patient at home [103]. The child should receive a personalized Asthma Action Plan (AAP) detailing ongoing therapy, and inhaled corticosteroids (ICS) should be initiated if not already prescribed. Scheduling follow-up visits is also essential to prevent future exacerbations and maintain quality of life [95,112].

Adherence to standardized criteria and guidelines ensures safety, reduces misclassification and treatment errors, and supports effective care for children with asthma. Strengthened collaboration between emergency services and outpatient care is vital to reduce the incidence of severe, life-threatening cases [115].

## 7. The Role of Education, Prevention, and the Management Plan After Discharge from the Emergency Department

While acute management and hospitalization are critical in pediatric asthma exacerbations, ongoing outpatient care is equally important to prevent recurrence and disease complications [116].

Education plays a pivotal role. Patients and caregivers must be taught proper disease control to avert future exacerbations [117]. Many remain unaware of the necessity of strict adherence to prescribed regimens. Studies show that approximately 29% of parents incorrectly believe inhaled corticosteroids (ICS) provide immediate bronchodilation for rescue use, while 24% report no knowledge of ICS at all [118]. Only 51% of children with chronic asthma use ICS daily. The study showed that a higher level of parental education was associated with more effective and correct use of ICS among asthmatic children. Asthma education employs various strategies, including innovative digital tools such as mobile video games. Most parents and children report a clear preference for such interactive methods over standard educational videos, citing improved understanding of asthma management and early recognition of warning signs for prompt intervention [119]. Parents must also understand the disease’s pathogenesis, identify triggers, and appreciate the critical importance of continuous medication use to lower the risk of future ED visits [120,121]. Common triggers of acute attacks include infections—frequent in younger children—and allergies, more prevalent in those over six years. Lack of controller therapy with ICS remains a significant risk factor for severe asthma episodes requiring emergency care [122]. Preventive recommendations therefore include appropriate immunizations, particularly seasonal inactivated influenza vaccination, which has demonstrated efficacy in reducing acute respiratory infections, asthma exacerbations, and related hospitalizations [123]. Pneumococcal vaccinations, which have recently gained an additional indication for children aged 6–18 years struggling with asthma are also recommended [124].

In the course of asthma prevention, for risk assessment, clinicians may use the Asthma Predictive Index (API) and Modified Asthma Predictive Index (mAPI). These tools evaluate factors such as annual episodes of wheezing, personal or family history of asthma, eczema, allergic rhinitis, non–cold-related wheeze, peripheral blood eosinophilia > 4%, and, additionally, in the modified scale, sensitivity to inhaled allergens or the occurrence of allergies to milk, eggs, or nuts. They are available in an easy and accessible form of online user-friendly calculators, which can facilitate rapid clinical evaluation of asthma risk during routine pediatric visits, enabling early intervention and prevention of full-blown disease [125,126]. Asthma, especially the pediatric-onset asthma is a strong predictor of reduced lung function and chronic airway obstruction, potentially progressing to COPD in adulthood, highlighting the importance of early prevention and annual spirometry in at-risk children [127].

Equally critical is a structured treatment plan with reliable adherence. Written Asthma Action Plans (WAAPs), based on current guidelines, are increasingly integrated with this challenge into clinical practice [128]. These plans teach patients to recognize early warning signs, perform home monitoring, and use medications correctly, thereby reducing unscheduled primary care or ED visits [129,130]. However, broader implementation and standardization can enhance their effectiveness and comprehension across the pediatric population [131].

There are also more detailed solutions covering the entire course of patient care, from discharge to the entire future course of the disease, including follow-up visits [132]. Some institutions have adopted this more comprehensive approaches such as the Safe Asthma Discharge Care Pathway (SADCP), piloted in parts of the United Kingdom. This program educates children and caregivers on symptom recognition, correct inhaler technique, and the importance of scheduled follow-ups and educational sessions [133]. Physician- or nurse-led training ensures proper inhaler use and full therapeutic dosing, improving clinical outcomes.

Community awareness initiatives also extend to schools, aiming to improve asthma care, ensure easier medication access, and facilitate early recognition of warning signs. Collaboration among caregivers, educators, and primary care physicians represents the most effective strategy for asthma prevention and for averting serious complications [134].

## 8. Conclusions

Acute asthma attacks are a common reason for pediatric patients to visit the hospital emergency department. Therefore, the key element of effective management is the diagnosis and assessment of the severity of asthma exacerbation using scales, most often the PRAM scale. Regardless of the severity of symptoms, the main drugs used in the emergency treatment of asthma exacerbations are SABA and oxygen therapy. The implementation of appropriate treatment and monitoring of patients in the post-attack period is the basis for therapeutic success. It should be emphasized that preventive measures and chronic treatment play a key role in the management of patients with asthma. The occurrence of asthma exacerbation indicates the failure of the current therapy, which necessitates its modification and the education of the patients’ parents [95].

## Figures and Tables

**Table 1:** Criteria for the severity of asthma exacerbation in children 6–11 years old according to the Global Initiative for Asthma 2025 (GINA 2025) [3].

Parameter	Exacerbation Severity		
	Mild/Moderate	Severe	Life-Threatening
Body position	Sitting > lying	Sits hunched forwards	
Speech	Sentences	Words	
Level of consciousness	Not agitated	agitated	Drowsy, confused
Respiratory rate	Increased	>30/min	
Accessory muscle in use	No	Yes	Paradoxical breathing
Pulse rate	100–120 bpm	>120 bpm	>120 bpm or bradycardia
SpO_2_ (on air)	90–95%	<90%	
PEF, % predicted or best	>50%	≤50%	Optionally

**Table 2:** Criteria for the severity of asthma exacerbation in children <5 years of age according to the Global Initiative for Asthma 2025 (GINA 2025) [13].

Symptoms	Exacerbation Severity	
	Mild	Severe or Life Threatening
Consciousness	-	Agitated, drowsy or confused
SaO_2_	>94%	<92%
Speech	Sentences	Words/ Unable to speak or drink
Respiratory rate	≤40/min	>40/min
Accessory muscle use	-	+
Pulse rate	<100/min	>180/min (0–3 years) >150/min (4–5 years)
Central cyanosis	-	+
Wheeze intensity	Variable	No wheezes

**Table 3:** The Pediatric Respiratory Assessment Measure (PRAM) [18,19,20].

Score	Intercostal Retractions	Suprasternal Retractions	Wheezing	Vesicular Breath Sounds	SpO_2_
0	none	none	none	present, normal	≥95%
1	-	-	expiratory wheeze	absent in the basal regions	92–94%
2	present	present	inspiratory and expiratory wheeze	generalized decreased breath sounds	<92%
3			audible without a stethoscope/silent chest with minimal air entry	minimal/absent breath sounds	

**Table 4:** Pediatric Asthma Severity Score PASS [20].

Score	Respiratory Effort	Wheezing	Prolonged Expiration
0	absent/mild	absent/mild	normal/mildly prolonged
1	moderate	moderate	moderately prolonged
2	severe	loud wheezing or absent wheezing due to poor air exchange	significantly prolonged

**Table 6:** Asthma Clinical Score (ACS) [28].

Score	Tachypnea (According to Age)	Oxygen Requirement (to Maintain SpO_2_ ≥92%)	Presence of Wheezing	Vesicular Breath Sound	Types of Retractions: (Nasal Flaring, Supraclavicular, Suprasternal, Intercostal, Subcostal)
0	no	room air	none	normal	none
1	yes	≤2 L/31%	end-expiratory or diffuse	moderate (diminished)	presence of one type of retraction
2		>2 L/31% and ≤4 L/50%	expiratory wheezes throughout the entire respiratory cycle	decreased	presence of two or more types of retractions
3		>4 L/50%	inspiratory and expiratory wheezes	absent breath sounds (silent chest)	
4			silent chest		

## Data Availability

No new data were created or analyzed in this study. Data sharing is not applicable to this article.

## Abbreviations

The following abbreviations are used in this manuscript:

ED	Emergency Department
ICU	Intensive Care Unit
PICU	Pediatric Intensive Care Unit
ICS	Inhaled Corticosteroids
GINA	Global Initiative for Asthma
RSV	Respiratory Syncytial Virus
PRAM	Paediatric Respiratory Assessment Measure
ASS	Asthma Severity Score
NAEPP	National Asthma Education and Prevention Program
SABA	Short- acting beta2-agonists
pMDI	Pressurised Metered Dose Inhaled
LABA	Long-acting beta2-agonists
OCS	Oral corticosteroids
SCS	Systemic corticosteroids
FEV1	Forced Expiratory Volume in 1 s
LTRAs	Leukotriene receptor antagonists
NIV	Non- invasive ventilation
IMV	Invasive mechanical ventilation
Spo2	Saturation of peripheal oxygen
NaCl	Sodium Chloride
PEFR	Peak Expiratory Flow Rate
CS	Corticosteroids
PEF	Peak Expiratory Flow
ER	Emergency Room
DALY	Disability-Adjusted Life-Years
BMI	Body mass index
NHIS	National Health Interview Survey
AAIRS	The Acute Asthma Intensity Research Score
PASS	Pediatric Asthma Severity Score
MPIS	Modified Pulmonary Index Score
ACS	Asthma Clinical Score
PFTs	Pulmonary Function Tests
AAP	Asthma Acting Plan
WAAPs	Written Asthma Action Plans
API	Asthma Predictive Index
mAPI	Modified Asthma Predictive Index
COPD	Chronic Obturatory Pulmonary Disease
SADCP	Safe Asthma Discharge Care Pathway
